# Placental Adaptation: What Can We Learn from Birthweight:Placental Weight Ratio?

**DOI:** 10.3389/fphys.2016.00028

**Published:** 2016-02-05

**Authors:** Christina E. Hayward, Samantha Lean, Colin P. Sibley, Rebecca L. Jones, Mark Wareing, Susan L. Greenwood, Mark R. Dilworth

**Affiliations:** ^1^Maternal and Fetal Health Research Centre, Institute of Human Development, University of ManchesterManchester, UK; ^2^Maternal and Fetal Health Research Centre, Manchester Academic Health Science Centre, St. Mary's Hospital, Central Manchester University Hospitals NHS Foundation TrustManchester, UK

**Keywords:** placenta, adaptation, birthweight, F:P ratio, FGR, nutrient transport

## Abstract

Appropriate fetal growth relies upon adequate placental nutrient transfer. Birthweight:placental weight ratio (BW:PW ratio) is often used as a proxy for placental efficiency, defined as the grams of fetus produced per gram placenta. An elevated BW:PW ratio in an appropriately grown fetus (small placenta) is assumed to be due to up-regulated placental nutrient transfer capacity i.e., a higher nutrient net flux per gram placenta. In fetal growth restriction (FGR), where a fetus fails to achieve its genetically pre-determined growth potential, placental weight and BW:PW ratio are often reduced which may indicate a placenta that fails to adapt its nutrient transfer capacity to compensate for its small size. This review considers the literature on BW:PW ratio in both large cohort studies of normal pregnancies and those studies offering insight into the relationship between BW:PW ratio and outcome measures including stillbirth, FGR, and subsequent postnatal consequences. The core of this review is the question of whether BW:PW ratio is truly indicative of altered placental efficiency, and whether changes in BW:PW ratio reflect those placentas which adapt their nutrient transfer according to their size. We consider this question using data from mice and humans, focusing upon studies that have measured the activity of the well characterized placental system A amino acid transporter, both in uncomplicated pregnancies and in FGR. Evidence suggests that BW:PW ratio is reduced both in FGR and in pregnancies resulting in a small for gestational age (SGA, birthweight < 10th centile) infant but this effect is more pronounced earlier in gestation (<28 weeks). In mice, there is a clear association between increased BW:PW ratio and increased placental system A activity. Additionally, there is good evidence in wild-type mice that small placentas upregulate placental nutrient transfer to prevent fetal undergrowth. In humans, this association between BW:PW ratio and placental system A activity is less clear and is worthy of further consideration, both in terms of system A and other placental nutrient transfer processes. This knowledge would help decide the value of measuring BW:PW ratio in terms of determining the risk of poor health outcomes, both in the neonatal period and long term.

## Introduction: birthweight:placental weight ratio and the importance of adequate placental nutrient transfer to the fetus

Appropriate nutrient provision to the fetus, via the placenta, is essential for optimal fetal growth. Placental dysfunction, and consequent reduced nutrient supply, is associated with fetal growth restriction (FGR), a condition in which the fetus fails to achieve its genetically pre-determined growth potential (Mahendran et al., 1993; Glazier et al., 1997; Baschat et al., 2007). The ability of the placenta to maintain adequate nutrient delivery to the fetus is determined by a number of factors including the total surface area of syncytiotrophoblast (transporting epithelium) available for exchange, and the abundance and activity of nutrient transporters [(Desforges and Sibley, 2010); placental nutrient transporter activity and expression is reviewed in detail elsewhere (Jones et al., 2007; Desforges and Sibley, 2010; Dilworth and Sibley, 2013)].

There is substantial evidence from studies in animals that the placenta regulates its nutrient transfer efficiency by morphological and functional adaptations which result in optimal fetal growth (Coan et al., 2008a; Fowden et al., 2009; Sandovici et al., 2012). Placental efficiency is commonly defined by birthweight:placental weight ratio (BW:PW ratio) i.e., the grams of fetus produced per gram placenta (Wilson and Ford, 2001). Placental efficiency thus acts as a proxy measure of how placental development/function has adapted to meet fetal nutritional requirements (Fowden et al., 2009). The morphological or functional placental adaptations (i.e., in nutrient transfer per gram placenta) can occur in response to either maternal or fetal cues that ultimately result in a change in placental efficiency (Fowden et al., 2009). It has been postulated that adaptations take place in order to maintain appropriate fetal growth, and that failure of placental adaptations may result in a fetus that is either too small or large with respect to its genetic growth potential (Sibley et al., 2010).

A pregnancy with a relatively large fetus, compared with the average fetal weight of the population, will have had a higher total net transfer of nutrients across the placenta over the course of gestation than the pregnancy with a relatively smaller fetus. An increase in placental efficiency, as evidenced by an increased BW:PW ratio, implies that nutrient transfer per gram placenta must have increased. Accordingly the reverse is true; a reduced BW:PW ratio suggests that nutrient transfer, per gram placenta, is reduced. Data from mouse studies suggest that this is indeed the case. In a wild-type (WT) mouse litter of normally grown fetuses near term, those fetuses with the lightest placentas within a litter are of an equivalent size to those from the heaviest placentas; accordingly the fetal:placental weight ratio (F:P ratio) is higher in the lightest placentas (Coan et al., 2008a). Importantly, in these lightest placentas within the WT litter, with an increased F:P ratio and placental efficiency, placental nutrient transfer (glucose and methylaminoisobutyric acid, a marker of system A amino acid transport) per gram placenta, is indeed also increased (Coan et al., 2008a). Thus, F:P ratio may be a useful predictor of those placentas that have adapted their nutrient transfer (per gram placenta). Additionally, it has been observed in a number of rodent models of FGR that F:P ratio is reduced (Jansson et al., 2006; Coan et al., 2008b, 2010; Kusinski et al., 2012), suggesting an inefficient placenta that failed to adapt its nutrient supply to meet the demands of the rapidly growing fetus and indeed, placental amino acid transport is reduced in all of these models (Aardema et al., 2001; Jansson et al., 2006; Coan et al., 2008b, 2010; Kusinski et al., 2012).

Less well understood is the association between placental efficiency, defined as above, and nutrient transfer in humans. Evidence showing that changes in BW:PW ratio were matched with similar alterations in placental nutrient transfer may help to identify those placentas adapting, or failing to adapt, their placental nutrient transfer according to fetal demand. This is important because, taking the example of FGR, a reduced BW:PW ratio may be indicative of inadequate placental nutrient transfer.

The review will consider two major themes. Firstly, in reviewing studies in women, we focus mainly upon large cohort studies measuring BW:PW ratio and offering insight into its relationship with outcome measures including stillbirth, abnormal fetal growth, and subsequent postnatal consequences. Secondly, this review will consider, in humans and mice, whether measures of placental nutrient transfer do in fact relate to measures of BW:PW ratio. As part of this, published data from our own *in vitro* studies of placental nutrient transfer in human will be collated and compared with other reported data in both the mouse and human, focusing on the extensively studied system A amino acid transporter system. Our goal is to help to ascertain whether BW:PW ratio is a fair proxy for placental efficiency in terms of nutrient transfer in human pregnancy and whether large deviations in BW:PW ratio, and particularly a reduced BW:PW ratio, may indicate those pregnancies with a failing placenta. It is these pregnancies that would likely benefit most from extra antenatal surveillance and therapeutic intervention in the future, particularly in the prevention of stillbirth (Heazell et al., 2015).

## Factors that influence BW:PW ratio and consequences of an altered BW:PW ratio for fetal wellbeing

Published BW:PW ratio measures are influenced by whether the placenta was weighed untrimmed or trimmed (removal of fetal cord and membranes) and this information is provided in the studies presented below. It is not intended for this review to be a systematic analysis of all studies quoting BW:PW ratio. Rather, the major focus is upon larger cohort studies that have examined important variables that may affect BW:PW ratio including sex, parity, ethnicity, and gestational age. Some studies present PW:BW ratio rather than BW:PW ratio but for consistency, all analysis and discussion of these studies has been related to BW:PW ratio.

There has been considerable interest in BW:PW ratio since the work of Barker and colleagues who suggested that those pregnancies with relatively large placentas relative to birthweight (low BW:PW ratio) are at increased risk of hypertension in adulthood (Barker et al., 1990). Similar findings have since been demonstrated in children; a low BW:PW ratio was associated with an increased risk of raised blood pressure (above 90th centile of blood pressures recorded in over 29,000 children) at 7-years of age (Hemachandra et al., 2006). Additionally, being in the lowest tertile of BW:PW ratio increases the relative risk of cardiovascular disease, and stroke in particular, in adulthood (Risnes et al., 2009). This leads to the suggestion that inefficient placentas, with supposed inadequate nutrient transfer, may be important in this programming of the fetus *in utero*, ultimately leading to increased risk of cardiovascular disease into adulthood.

BW:PW ratio increases across gestation as placental growth slows and fetal growth accelerates during the third trimester (Molteni et al., 1978). BW:PW ratio doubles from week 24 of pregnancy to term (38–40 weeks; Almog et al., 2011; Wallace et al., 2013). A number of large cohort studies have determined BW:PW ratio in pregnancies with normal outcome (Burkhardt et al., 2006; Almog et al., 2011; Wallace et al., 2013) and birthweight is 5–7 times greater than placental weight at term. In a comprehensive study based in Aberdeen, UK, Wallace et al. constructed placental weight (untrimmed) and BW:PW ratio centiles for over 89,000 pregnancies (Wallace et al., 2013). Separate centile charts were produced according to parity and fetal sex and split to account for gestational age (from 24 to 43 weeks). Placental weight and BW:PW ratio centiles were presented and showed that BW:PW ratio was higher in males but parity had no effect. This is in contrast to placental weight; multiparous women had significantly heavier placentas than nulliparous women. Additionally, the placentas of males were significantly heavier than those of females but this effect of sex was less marked than the effect of parity.

This splitting of BW:PW ratio by sex has also been documented in another large cohort study in Montreal, Canada (Almog et al., 2011) which calculated centiles similar to those in the study by Wallace et al.; the effects of parity were not reported in this study but centiles for placental weights (trimmed) were also produced for twin pregnancies. This study did not compare BW:PW ratio between males and females statistically but did show that the median placental weight at term was higher for males (679 g) than for females (668 g). Additionally, there was a correlation between gestational age and BW:PW ratio in both females and males. The authors concluded that these data should act as a reference tool for North American populations, but urged caution if used to compare against other cohorts, particularly when considering the different ethnicities that may be present within these different populations.

One of the largest studies quoting BW:PW ratio (untrimmed placental weight) examined over 500,000 singleton births in Norway (Haavaldsen et al., 2013); this encompassed all births between 1999 and 2008 and included confounders such as pre-eclampsia, diabetes, and those pregnancies conceived following assisted reproductive technology. This study considered the relative risk of fetal death in the lowest and highest quartiles of BW:PW ratio in both pre-term (23–36 weeks) and term (37–42 weeks) pregnancies. Values were adjusted for maternal characteristics including age, parity and smoking status and the other confounders detailed above. The study demonstrated elevated odds ratios (OR) for fetal death in both the lowest (OR 1.79, 95%CI 1.55–2.08) and highest (1.67, 1.44–1.94) BW:PW ratio quartiles in the pre-term group; in the term group, only the highest BW:PW ratio centile demonstrated an increased OR (1.76, 1.50–2.06) compared to the middle centiles. This suggests that, in this Norwegian population, a smaller placenta relative to fetal size, hypothesized to be a more efficient placenta in terms of nutrient transfer, was a risk factor for fetal death at term.

Williams and colleagues assessed BW:PW ratio in over 2500 pregnancies in Perth, Australia, and showed that both placental weight (untrimmed) and birthweight were better independent predictors of newborn morphometrics (such as abdominal and head circumference, ponderal index) than BW:PW ratio and thus concluded that BW:PW ratio was a poor proxy of fetal growth (Williams et al., 1997). This emphasizes a seemingly obvious, but important point, that in order to fully understand the significance of BW:PW ratio in relation to placental efficiency, it is essential to consider it in the context of the actual birthweight and placental weight (or birthweight/placental weight centile), since a normal BW:PW ratio could be the result of a birthweight and placental weight that are both normal, both high, or both low (Williams et al., 1997; Hutcheon et al., 2012). The authors also commented on the importance of accurate gestational ages when considering BW:PW ratio (Williams et al., 1997).

Shehata and colleagues studied 19,000 women in Canada delivering infants appropriate for gestational age (AGA) at term, grouped into centiles of BW:PW ratio. They demonstrated that a BW:PW ratio below the 10th centile presented with a greater risk of both NICU admission (OR 2.54, 2.01–3.20) and an apgar score below 7 (OR 1.82, 1.21–2.73) whilst a high (above 90th centile) BW:PW ratio appeared to reduce the risk of NICU admission (OR 0.59, 0.38–0.90; Shehata et al., 2011). This confirmed a study by Lao and Wong demonstrating again in AGA only, that infants with a low BW:PW ratio (greater than 1 SD below the mean of the population, untrimmed placentas) presented with an increased risk of a low apgar score (<7; Lao and Wong, 2001). These data suggest that, even in a seemingly lower risk AGA population, BW:PW ratio may be indicative of immediate and longer term health risks for an individual.

Individually, a number of these studies act as a useful reference tool for comparisons of BW:PW ratio against populations with similar characteristics (e.g., ethnicity). Collectively they demonstrate some of the factors that influence BW:PW ratio including gestational age, ethnicity, parity, and fetal sex, and begin to outline the links between an altered BW:PW ratio and risk of short and long-term health. The next section discusses the concept of placental adaptation and considers whether BW:PW ratio can be used to indicate those placentas that adapt nutrient transfer in relation to placental size.

## Evidence of placental adaptation in normal pregnancy

**Figure 1:**
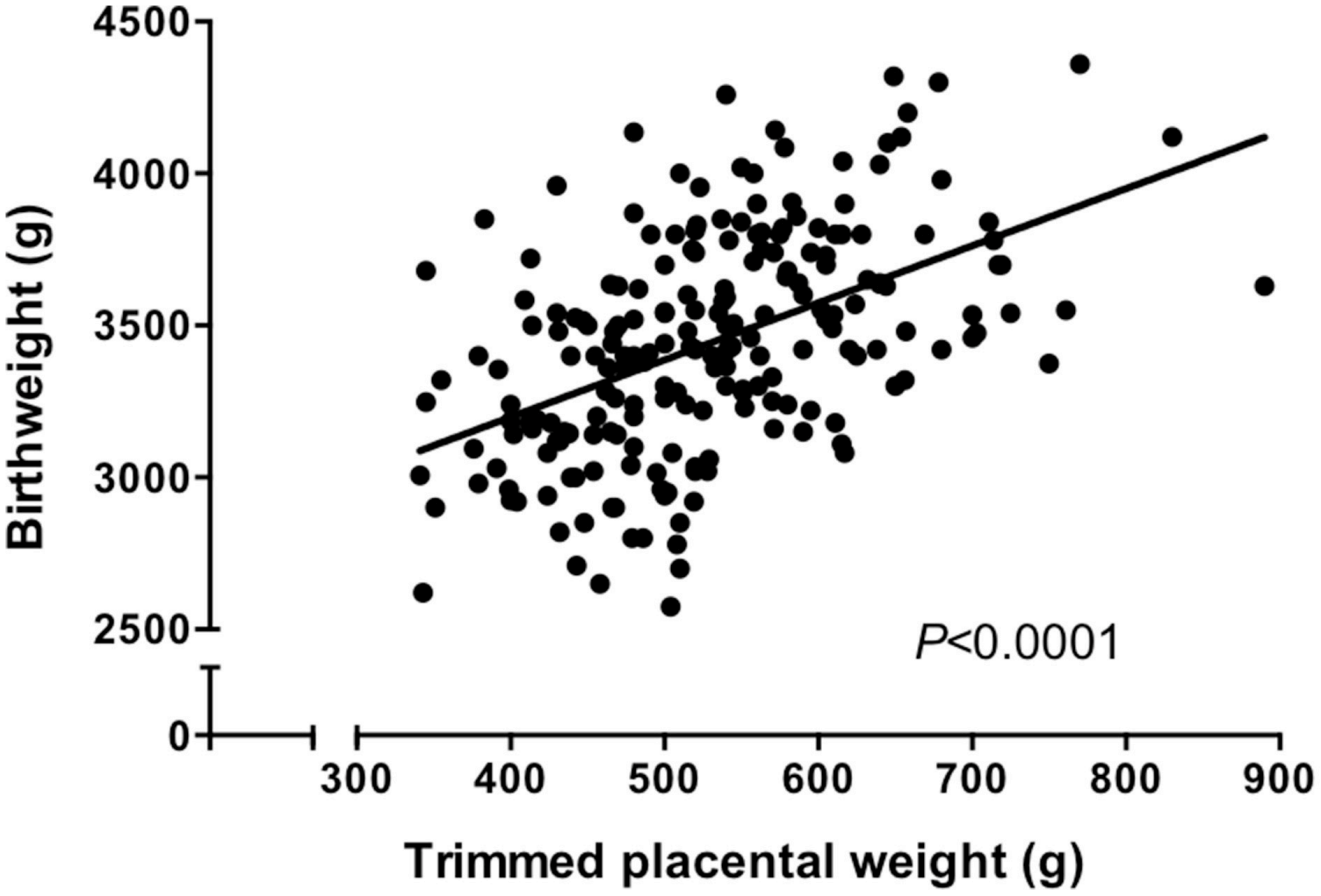
**Trimmed placental weight vs. birthweight in an appropriate for gestational age (>10th, < 90th centile of IBCs) population**. There was a significant correlation between placental weight and birthweight (*P* < 0.0001, Spearman correlation). *N* = 210. Data represents a collation of our previously published data (Ditchfield et al., 2010, 2015; Hayward et al., 2012, 2013; Brereton et al., 2013; Coleman et al., 2013; Lean et al., 2014; Jones et al., 2015; Mills et al., 2015) and represents a well characterized cohort all of whom delivered at St Mary's Hospital, Manchester.

It is well established that there is a positive correlation between placental weight and birthweight in human pregnancy (Winick et al., 1967; Crawford et al., 1987; Thame et al., 2001, 2004; see also Figure 1). This suggests that the size of the placenta dictates the size of the fetus, an assumption supported experimentally by studies in animals; it was shown over 30 years ago that sheep in which placental size was reduced by removal of endometrial caruncles had growth restricted lambs (Robinson et al., 1979; Harding et al., 1985). The correlation between placental weight and birthweight suggests that BW:PW ratio should be fairly constant between different women. Thus, large deviations in BW:PW ratio from the norm are indicative of either a baby that is relatively larger, or smaller, than would be expected from a placenta of a certain size, or conversely, a placenta that is relatively larger, or smaller than would be expected from a baby of a certain size. This may indicate those placentas that are adapting their nutrient supply to compensate for a relatively small or large placental size.

Placental adaptation has been the subject of previous reviews (Fowden et al., 2009; Sibley et al., 2010; Sandovici et al., 2012) and the concept is summarized here. In normal pregnancy, the growth of the fetus is by definition, within the normal range of fetal weights and net placental nutrient transfer over the course of gestation is sufficient to meet fetal nutrient requirements. This, however, is unlikely to tell the whole story and there may have been specific points during normal pregnancy where net placental nutrient transfer was insufficient to meet fetal nutrient demand. This scenario would lead to either undergrowth or overgrowth of the fetus unless the placenta was able to adapt its nutrient transfer relative to fetal demand. A placenta could adapt by increasing, or decreasing, the transfer of nutrients per gram. This may be through a combination of morphological and functional adaptations. It has been suggested that the ability of the placenta to adapt according to fetal nutrient demand prevents FGR or fetal macrosomia occurring more frequently (Sibley et al., 2010). By contrast, in cases of abnormal fetal growth, it is hypothesized that the placenta fails to adapt its nutrient transfer according to fetal nutrient demand, thus resulting in a fetus that is either too small or large.

Some of the strongest evidence for placental adaptation and of a direct link between changes in BW:PW ratio comes from a study by Coan and colleagues in the mouse (Coan et al., 2008a), which we touched on in the introduction to this review. In this study, F:P ratio was assessed in WT mice at both embryonic day (E) 16 and E19 (term = E20). As in humans, F:P ratio increases across gestation but this difference is even more pronounced in mice; F:P ratio rises from around 4 at E16 to ~11–14 at E19. This reflects the rapid fetal growth rate, the stagnation of placental growth and the increased rates of nutrient transfer seen across these time points (Coan et al., 2008a; Dilworth et al., 2011). In the study by Coan et al., natural variations in placental size were investigated by examining the lightest vs. heaviest placenta within a WT litter and assessing fetal weight and F:P ratio from these individuals. The authors reported a reduction in placental weight of around 30% between lightest and heaviest placentas at both gestational ages. However, fetal weight was significantly lower in fetuses from the lightest vs. heaviest placentas at E16; by E19, the day before term, this difference was no longer apparent (Coan et al., 2008a). To explore the placental mechanisms underpinning this “normalization” of fetal weight between E16 and E19, the authors examined whether there were any morphological or functional changes in the lightest vs. heaviest placentas between these time-points. Independent of whether it was the lightest or heaviest placenta within the litter, there was a significant increase in mean surface area of the maternal and fetal-facing surfaces of the interhaemal trophoblast membrane between E16 and E19 with an accompanying reduction in mean thickness of this membrane. Together, these resulted in a significant increase in the theoretical diffusion capacity at E19. When examining the different zones of the mouse placenta, it was also observed that the % volume fraction of the labyrinthine zone (exchange layer of the mouse placenta) was relatively increased at E19 vs. E16. These changes represent normal morphological changes that occur in the mouse placenta as pregnancy progresses. When comparing lightest vs. heaviest placentas, there were no differences in mean surface area or thickness of the interhaemal trophoblast membrane at E16 or E19; thus the theoretical diffusion capacity was unaltered. Lightest placentas demonstrated a reduced size of junctional zone (suggested to play an important endocrine role) and decidual basalis layer at E16 with no difference in the size of the labyrinthine zone. At E19, the reverse was true; the labyrinthine zone was reduced in the lightest vs. heaviest placentas with no change in the junctional zone or decidual basalis. When expressed as % of the total placental size, the only differences observed were a reduced labyrinthine and junctional zone at E16 in the lightest vs. heaviest placentas, indicating that these reductions were more than simply a proportional reduction appropriate to the reduced placental size.

To examine the evidence for functional adaptation within these placentas, Coan and colleagues assessed placental nutrient transfer between E16 and E19, focusing upon markers of passive permeability (^14^C-inulin), facilitated diffusion (^14^C-glucose), and secondary active transport (^14^C-MeAIB). MeAIB is a non-metabolizable substrate used as a proxy for the system A amino acid transporter. These studies were carried out *in vivo* following injection of radiolabeled tracers into the maternal circulation and subsequent fetal radiolabel counts, allowing calculation of unidirectional maternofetal clearance (K_mf_). The authors found no difference in K_mf_ (per g placental tissue) of ^14^C- inulin or glucose between lightest and heaviest placentas at E16 but K_mf_ of ^14^C-MeAIB was significantly upregulated in the lightest placentas. At E19, K_mf_ of ^14^C- glucose and MeAIB were significantly increased in lightest vs. heaviest placentas whilst ^14^C^−^inulin demonstrated no difference. The authors concluded that there were morphological and functional changes within the placenta in response to changes in placental size and that these changes, hypothesized to be adaptations, may occur in response to fetal nutrient demand. These changes were largely morphological at E16 and functional at E19 and also suggest that large and small placentas adopt different strategies to maintain appropriate fetal growth. A study comparing lightest and heaviest placentas with those around mean weight within the same litter would help to corroborate this further.

These data provide evidence of placental adaptation, in terms of specific facets of nutrient transfer capacity, in normal mouse pregnancy. Studies specifically investigating this in human are largely limited to placental system A amino acid transporter activity, and will be discussed later in this review. Firstly, it is important to comment on the studies that have investigated BW:PW ratio in complications of pregnancy.

## Birth weight:placental weight ratio and placental adaptation in complications of pregnancy

A significant reduction in BW:PW ratio may be indicative of placental dysfunction, the major cause of FGR, and a number of studies report reduced BW:PW ratio in FGR and SGA populations (Bortolus et al., 1998; Lurie et al., 1999; Lackman et al., 2001; Macdonald et al., 2014a,b). Molteni and colleagues charted gestational changes in placental and fetal weight infants destined to be SGA (BW < 10th centile), AGA (BW > 10th, < 90th centile) or large for gestational age (LGA, BW > 90th centile) and showed that placentas of SGA babies do not gain weight after 36 weeks gestation whereas placental weight in AGA and LGA babies continues to rise until 42 weeks (Molteni et al., 1978). In a comprehensive study of birthweight and untrimmed placental weight in over 40,000 pregnancies, Macdonald and colleagues found that SGA infants had lower BW:PW ratio compared to AGA infants earlier in pregnancy (24–28 weeks) but that this difference was not as great at term, with a small reduction in BW:PW ratio in females only (Macdonald et al., 2014a). The finding that the differences in BW:PW ratio between SGA, AGA, and LGA groups is more marked earlier in gestation has been observed previously (Molteni et al., 1978; Imada et al., 2012). The decreased BW:PW ratio earlier in gestation in the SGA group (Macdonald et al., 2014a) would be indicative of a fetus that is undergrown relative to placental size and thus implies functional inefficiency of the placenta, which may imply a pathological process.

Macdonald et al. suggested that BW:PW ratio may be a candidate to aid detection of SGA fetuses early in pregnancy; this would be particularly attractive in terms of identifying those pregnancies complicated by FGR, but would rely on accurate estimation of fetal and placental size following ultrasonography. The authors also suggest that this decreased BW:PW ratio in the SGA group early in gestation, when compared with the placental weight and birthweight centiles across gestation, is likely due to altered placental function rather than size, which again hints toward a pathological process (Macdonald et al., 2014a). Following this study, the authors investigated the links between obstetric conditions associated with hypoxia and BW:PW ratio (using untrimmed placental weight data), separating their data into three groups (<33 weeks gestation, 33–37 weeks and >37 weeks) given the effect of gestation on BW:PW ratio (Macdonald et al., 2014b). The authors found that obstetric conditions associated with fetal hypoxia, both due to pre-placental hypoxia (e.g., maternal anemia, smoking) or placental hypoxia [e.g., preeclampsia (PE), SGA, placenta previa] were more likely to be associated with a BW:PW ratio below the 10th centile (Macdonald et al., 2014b) with a higher likelihood of a reduced BW:PW ratio in PE before 33 weeks and SGA at term. Maternal anemia has been previously linked with low BW:PW ratio (Godfrey et al., 1991).

Lackman et al. demonstrated that babies below the 3rd centile of birthweights, classed as FGR in this study, had a significantly reduced BW:PW ratio, based upon untrimmed placental weights, compared with AGA pregnancies near term. This significant reduction was also seen in babies between the 3rd and 10th birthweight centiles, which was referred to as “borderline FGR” in this study (Lackman et al., 2001). A study in Israel by Lurie et al. demonstrated that babies born at term below 2.5 kg demonstrated the lowest BW:PW ratio (untrimmed placental weight) although this was based upon birthweight rather than individualized birthweight centiles as used in most other studies. This study also reinforced the importance of gestational age upon BW:PW ratio and showed that even at term (37–42 weeks) there were subtle changes in BW:PW ratio week on week, which complicates analyses between individuals (Lurie et al., 1999). In a Finnish cohort of over 16,000 women, pregnancies with a SGA infant (below 10th birthweight centile) had a small, but statistically significantly reduced BW:PW ratio compared with AGA (untrimmed placental weights) and taking into account gestational age (Heinonen et al., 2001). When comparing AGA with SGA individuals that were matched for birthweight, it was the SGA group that demonstrated an increased BW:PW ratio, at odds to many other studies but in support of the study by Heinonen and colleagues that showed a small, but significantly elevated BW:PW ratio in SGA vs. AGA infants (Heinonen et al., 2001).

Lao and colleagues examined BW:PW ratio in 470 pregnancies in Hong Kong and compared AGA, SGA, and LGA groups. They showed that BW:PW ratio was significantly lower in the SGA group compared with AGA with a trend toward an increased BW:PW ratio in the LGA group (Lao and Wong, 1996). Placental (untrimmed) weight in the SGA group however was not significantly different from the AGA group, indicating that this reduction in BW:PW ratio was primarily due to the reduced birthweight in the SGA group; the authors suggested that this may be due to limitations on fetal growth in order to maintain placental function as has been previously suggested (Barker et al., 1993). To examine this SGA group in further detail, the same authors carried out a retrospective study examining 252 cases of SGA only. These cases were split into three groups according to their BW:PW ratio, greater than 1SD above (high) or below (low) the mean BW:PW ratio, or within 1 SD of the mean (normal) (Lao and Wong, 1999). It was demonstrated that BW:PW ratio in the low group was reduced due to both a larger placenta (weighed untrimmed) and reduced birthweight compared with the normal group. These differences were independent of any alterations in maternal characteristics with the exception of a trend (not significant) toward increased smoking within the low group. Due to the increased neonatal morbidity in this sub-group, the authors suggested that this SGA group with a low BW:PW ratio may be more indicative of FGR. These findings indicate the importance of trying to classify individual sub-groups within an SGA cohort.

Manipulation of both fetal and placental growth in mice, for example by the production of genetic knock out or transgenic mice, results in alterations in F:P ratio. Mice with deletion of the endothelial nitric oxide synthase (eNOS^−∕−^) gene have reduced uterine blood flow velocity and reduced fetal weight with no difference in placental weight; thus F:P ratio is reduced in this model of FGR (Kulandavelu et al., 2012; Kusinski et al., 2012). Manipulation of the insulin-like growth factor 2 (*Igf2*) axis in mice also results in altered fetal growth. Mice with global deletion of *Igf2* have severely reduced fetal and placental weight and a reduced F:P ratio near term (Constância et al., 2005). In this model, nutrient delivery to the fetus (glucose and MeAIB) is reduced. It has been suggested that these mice do not adapt their placental nutrient transfer according to reduced placental size and thus FGR ensues. However, in placental specific *Igf2* knockout (P0) mice, in which fetal levels of *Igf2* are unaffected, there is evidence both of placental adaptation and of its failure (Constância et al., 2002, 2005). At embryonic day (E) 16, 4 days prior to term, there is reduced placental weight in P0 pups (30% smaller than WT) with fetal growth only reduced by 4% compared with WT. By E19, FGR (25% reduction in fetal weight vs. WT) has ensued with placental weight remaining reduced in P0 compared with WT siblings. This led to the hypothesis that there were functional adaptations within the smaller P0 placenta occurring at E16 which prevented fetal undergrowth, and that failure of this adaptation resulted in FGR at E19. To test this hypothesis, placental nutrient transfer (^51^Cr-EDTA, a marker of passive permeability, and ^14^C-MeAIB) was assessed in a similar manner to the study on WT mice by Coan and colleagues and described above (Coan et al., 2008a). It was demonstrated that placental transfer of MeAIB, per gram of placenta, was upregulated at E16; by E19 this upregulation no longer occurred and delivery of MeAIB, as measured in the P0 fetus, was reduced vs. WT (Constância et al., 2002). Placental transfer of ^51^Cr-EDTA was comparable between P0 and WT at E16, but reduced in P0 at E19 (Constância et al., 2002), an observation confirmed in a separate study and also demonstrated for ^14^C-mannitol, another marker of passive permeability (Sibley et al., 2004). Interestingly, placental transfer of ^14^C-glucose, per gram of placenta, was comparable between P0 and WT at both E16 and E19 (Constância et al., 2005). Despite this observed FGR, F:P ratio in P0 knockout mice remains higher at E19; indeed activity of some nutrient transport systems such as glucose and calcium still appear to be increased at E19 in the placentas of these mice (Constância et al., 2005; Dilworth et al., 2010). This upregulation in calcium transport in P0 placentas supports data in humans that plasma membrane calcium ATPase, located on the fetal facing basal plasma membrane of the syncytiotrophoblast and responsible for transporting Ca^2+^ to the fetus against a concentration gradient, is upregulated in human FGR (Strid et al., 2003). This remains the only placental nutrient transporter, thus far, to be shown to be upregulated in human FGR and provides evidence to suggest that, for some nutrient transport systems, adaptations in placental nutrient transfer may also be present in cases of abnormal fetal growth.

Placental adaptation in relation to overgrowth has also been studied in mice. Increasing levels of fetal and placental IGF2, by deleting the *H19* region that incorporates the *H19/Igf2* imprinting control region, resulted in placental and fetal overgrowth (Angiolini et al., 2011). Despite this fetal overgrowth, F:P ratio was reduced in H19 knockout mice at both E16 and E19. This was accompanied by reductions in placental nutrient transfer capacity; markers of passive permeability, glucose and system A amino acid transport were all reduced per gram placenta, and hints toward mechanisms to limit the extent of the fetal overgrowth; i.e., further evidence of a placenta that is demonstrating adaptation. These data in mice provide strong evidence of placental adaptation in relation to size, and to F:P ratio, in both FGR and fetal overgrowth.

BW:PW ratio has been measured in other complications of pregnancy in humans, including gestational diabetes mellitus (GDM) and pre-eclampsia (PE). Lao and colleagues performed a retrospective study on women with GDM or impaired glucose tolerance (IGT), both of which were controlled through diet (Lao et al., 1997). These women were compared with women with similar risk factors for GDM/IGT, but who demonstrated normal glucose tolerance. BW:PW ratio (untrimmed placental weights) was decreased in both GDM and IGT groups compared with controls, but this may have been confounded by differences in gestational ages between groups. In a study in Korea, Kim and colleagues suggested that BW:PW ratio was increased in GDM although numbers were low (33 GDM v 82 controls; Kim et al., 2014). In this same study, the authors also demonstrated that BW:PW ratio was reduced in PE vs. controls; the effect was seen independent of whether PE presented with or without FGR (Kim et al., 2014) and was complicated by the fact that the PE group tended to deliver around 3 weeks earlier on average.

Taken together, these studies suggest that there are deviations in BW:PW ratio when comparing complicated pregnancies with normal outcomes. In particular, the weight of evidence suggests that BW:PW ratio is reduced in both SGA and FGR, with the caveat that this is more profound earlier in gestation. To assess whether changes in BW:PW ratio, and thus placental efficiency, are mirrored by changes in placental nutrient transfer capacity in human pregnancy, as seen in the mouse, the following section considers associations between the activity of the placental system A amino acid transporter system and BW:PW ratio.

## System a amino acid transporter activity in relation to BW:PW; microvillous plasma membrane vesicle studies

System A is a transporter system present on both the microvillous and basal plasma membranes of the syncytiotrophoblast of the placenta that transports small, neutral amino acids, such as alanine, serine, and glycine, in a sodium dependent manner (Mahendran et al., 1993; Glazier et al., 1997). System A consists of three isoforms within the placenta. These isoforms are the Na^+^-coupled neutral amino acid transporters (SNATS) 1, 2, and 4, encoded by SLC38A1, 2 and 4, respectively (Mackenzie and Erickson, 2004; Desforges et al., 2006). Both because of the important contribution the substrates of this transporter make to fetal growth and because its placental activity is relatively straightforward to measure, a number of studies have related placental system A activity to fetal and placental growth. In SGA and FGR infants, reduced placental size is frequently observed and system A activity (per mg placental protein) also has been shown to be reduced compared with AGA populations, particularly in pre-term groups with severe FGR (Mahendran et al., 1993; Glazier et al., 1997; Harrington et al., 1999; Jansson et al., 2002a; Shibata et al., 2008). Likewise, data in mice and rats suggest that placental system A amino acid transport is dysregulated in genetic and dietary models of FGR (Malandro et al., 1996; Constância et al., 2002; Jansson et al., 2006; Kusinski et al., 2012; Stanley et al., 2012). These studies appear to indicate the importance of placental system A transport in terms of fetal growth. Placental system A activity has also been measured in a number of studies investigating uncomplicated pregnancies and these studies afford the opportunity to examine whether system A activity is normally associated with altered BW:PW ratio, and thus placental efficiency, as demonstrated in the mouse (Constância et al., 2005; Coan et al., 2008a; Dilworth et al., 2010, 2011).

Godfrey et al. (1998) investigated the activity of the system A transporter in the syncytiotrophoblast microvillous plasma membrane (MVM) in relation to birthweight in uncomplicated pregnancy. System A activity was estimated by determining the sodium-dependent component of ^14^C-MeAIB uptake into MVM vesicles (relatively pure spheres of plasma membrane with no intracellular machinery) (Glazier and Sibley, 2006) isolated from these placentas. MeAIB, a non-metabolizable substrate, is used as a proxy for system A activity. Whilst there are sodium-independent mechanisms of MeAIB uptake, not attributable to system A, the sodium-dependent component of ^14^C-MeAIB uptake is a measure of system A activity. The authors observed that system A activity (nmol/mg membrane protein) was increased in the placentas of the smallest babies (Godfrey et al., 1998), which was somewhat surprising given the data showing that placental system A activity, per mg protein, is reduced in FGR and SGA (Mahendran et al., 1993; Glazier et al., 1996, 1997; Shibata et al., 2008). This increased system A activity was suggested to be a compensatory mechanism by the placentas of these smaller infants within the normal birthweight range in response to increased fetal nutrient demand. This is of course akin to what was found in mice some years later: as discussed in detail above, K_mf_ of ^14^C-MeAIB was upregulated in the lightest vs. heaviest placentas of WT mice both at a time point when fetal growth was reduced (E16) and normalized (E19) (Coan et al., 2008a). K_mf_ of ^14^C-MeAIB was also upregulated in the pathologically small *Igf2* P0 knockout mouse placenta 4 days prior to term at a time when fetal growth was reduced (but only by 4%), vs. wild-type (Constância et al., 2002).

In the study by Godfrey et al. (1998), the relationship between system A activity and BW:PW ratio was not reported. However, in a separate study by Harrington et al., also employing the MVM vesicle technique, a negative correlation was observed between placental ratio (PW:BW ratio) and system A activity in a well defined AGA population, which infers a positive correlation between BW:PW ratio and system A activity (Harrington et al., 1999). This is consistent with data in WT mice *in vivo* showing that increased F:P is associated with increased unidirectional maternofetal clearance of MeAIB per gram placenta (Coan et al., 2008a). Together, this study of system A activity in MVM vesicles from human placenta and studies of placental MeAIB transfer by system A in the mouse are consistent in showing a positive relationship with BW:PW ratio (F:P ratio in mice) and thus placental efficiency. In the study by Harrington and colleagues, there was no correlation between system A activity and birthweight (Harrington et al., 1999), which is at odds with the negative association suggested by Godfrey et al. (1998). Whilst both of these studies employed MVM vesicles, Harrington et al. investigated a defined AGA population (10th–90th centiles based upon individualized birthweight centiles, IBCs, which take into account maternal characteristics such as BMI, ethnicity and parity, and gestational age) whereas Godfrey et al. defined normal by birthweight alone and did not employ IBCs. In a study in Sweden, Jansson and colleagues found a positive correlation between system A activity in MVM vesicles (pmol/mg protein) and birthweight (Jansson et al., 2013). The Jansson study included data from women with a normal body mass index (BMI) and from women presenting with obesity (BMI > 30). Despite the fact that there was no effect of maternal BMI on system A activity, the presence of women with obesity in this dataset means caution is required when making comparisons with the data of Godfrey and Harrington. The reasons for the reports of negative (Godfrey), no relationship (Harrington) and positive associations (Jansson) between MVM system A activity and BW are not obvious, and this along with the paucity of data relating system A activity to BW:PW ratio in human pregnancy led us to examine these associations further using data from our laboratory, as discussed in the section that follows.

## System a amino acid transporter activity in relation to BW:PW; placental fragment studies in manchester

Since we have performed a number of recent studies investigating system A activity in placental villous fragments (Ditchfield et al., 2010; Hayward et al., 2012; Lean et al., 2014), we took the opportunity to collate this data and re-examine the relationship between system A activity, BW, PW, and BW:PW ratio in pregnancies resulting in AGA birth. All placental tissue was acquired from women attending St Mary's Hospital, Manchester for their antenatal care and following informed consent, under ethical approval by the North West Haydock Park Research Ethics Committee (08/H1010/05) and the University of Manchester Ethics Committee. Procedures were followed in accordance with institutional guidelines and conformed to the standards set by the latest revision of the *Declaration of Helsinki*.

Placental villous fragments represent a different preparation to the studies considered above which employed MVM vesicles (Godfrey et al., 1998; Jansson et al., 2013) and the preparation has been described in detail elsewhere (Greenwood and Sibley, 2006). Unlike MVM vesicles, villous fragments retain cellular machinery. Fragments were incubated for set time periods in ^14^C-MeAIB and radiolabel accumulation (uptake) was measured in relation to the protein content of the villous tissue. A time-course was adopted over which MeAIB uptake into the fragments was linear (typically 30–120 min incubation with MeAIB) to ensure that system A activity was assessed at initial rate (Greenwood and Sibley, 2006). During their dissection from the placenta, villous fragments are subject to damage that could allow non-specific (i.e., not via system A) diffusion of ^14^C-MeAIB into the tissue. To account for this, utilizing the sodium-dependent nature of system A transport, uptake of ^14^C-MeAIB into villous fragments was carried out both in the presence and absence of sodium, enabling estimation of system A activity as the sodium-dependent component of total uptake. In the fragment preparation, sodium-dependent ^14^C-MeAIB uptake was assumed to represent system A activity in the MVM of the syncytiotrophoblast (Greenwood and Sibley, 2006).

**Figure 2:**
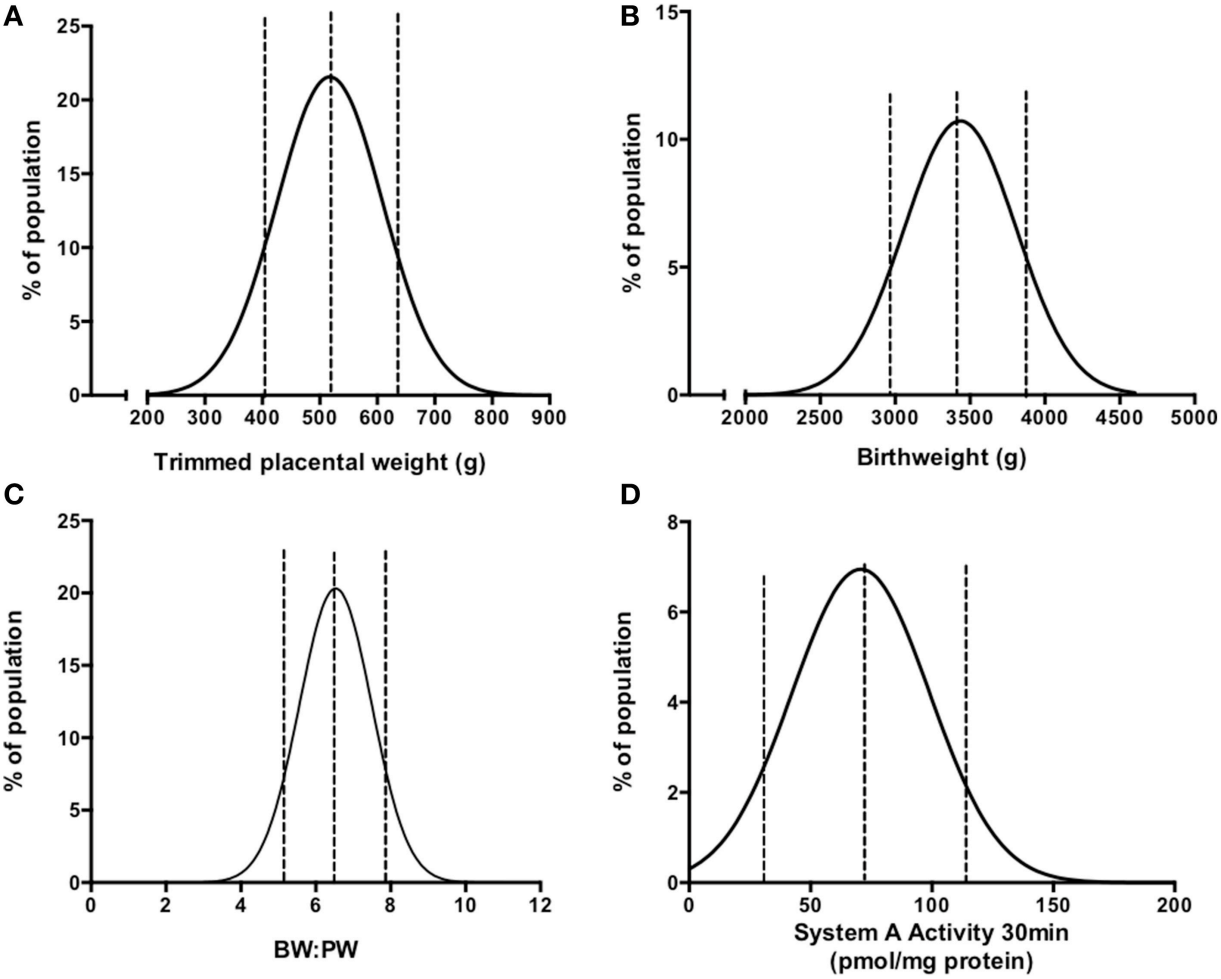
**Frequency (%) distributions for trimmed placental weight (A), birthweight (B), BW:PW ratio (C), and placental system A activity (D) in an AGA population following collation of previously published data (Ditchfield et al., 2010, 2015; Hayward et al., 2012, 2013; Brereton et al., 2013; Coleman et al., 2013; Lean et al., 2014; Jones et al., 2015; Mills et al., 2015)**. Dotted lines denote the 10th, 50th, and 90th centile. Actual values are as follows (10th, 50th, 90th centile); For **(A)** 408, 520, and 642 g; **(B)** 2964, 3430, and 3896 g; **(C)** 5.2, 6.6, 7.5; **(D)** 33.2, 74.4, 115.6 pmol/mg protein. *R*^2^-values of best fit to Gaussian distribution were as follows **(A)** 0.989, **(B)** 0.903, **(C)** 0.990, **(D)** 0.893. *N* = 210 for **(A–C)** and *N* = 60 for **(D)**.

Prior to carrying out this system A re-analysis, we also took the opportunity to examine a correlation between birthweight and placental weight from those pregnancies for which we had system A activity data (*N* = 60; Ditchfield et al., 2010; Hayward et al., 2012; Lean et al., 2014). These data were combined with those from other placentas of AGA pregnancies for which we have previously published trimmed placental weight and birthweight data (total *N* = 210; Brereton et al., 2013; Coleman et al., 2013; Hayward et al., 2013; Ditchfield et al., 2015; Jones et al., 2015; Mills et al., 2015). All of these pregnancies were classed as normal, with fetal weights between the 10th and 90th centile of IBCs. Additionally, pregnancies with confounders such as PE and BMI over 30 were excluded. In common with previous population studies (Thame et al., 2001, 2004), we demonstrated a significant positive correlation between placental weight and birthweight (Figure 1). Frequency (%) distribution curves for birthweight, placental weight, and BW:PW ratio from this population are shown in Figure 2 and afford the opportunity to relate system A activity to our own population centiles for birthweight, placental weight, and BW:PW ratio, as well as acting as a useful reference for future comparative studies.

Within this population, system A activity was previously measured using placental villous fragments (Ditchfield et al., 2010; Hayward et al., 2012; Lean et al., 2014) on 60 placentas and was re-plotted as a frequency (%) distribution, shown in Figure 2D. These studies are a collective of a number of studies in our laboratory (Ditchfield et al., 2010; Hayward et al., 2012; Lean et al., 2014), all of which assessed system A activity using the same methodology as described above and in (Greenwood and Sibley, 2006). As mentioned, a number of confounders suggested to affect placental system A activity in women, such as obesity (Jansson et al., 2013), GDM (Jansson et al., 2002b) “young” (teenage; Hayward et al., 2012), and advanced (>40 years of age) (Lean et al., 2014) maternal age were excluded and thus these 60 individuals represent a well defined “normal” cohort. All studies assessed uptake across a range of time points and in all cases ^14^C-MeAIB uptake was linear up to 30 min and this time point was selected to relate system A activity to birthweight, placental weight and BW:PW ratio.

**Figure 3:**
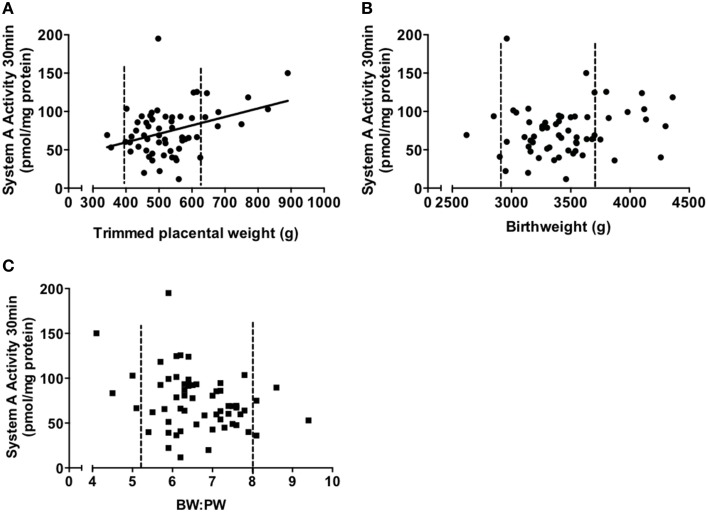
**Correlations of system A activity (pmol/mg protein) vs. trimmed placental weight (A), birthweight (B), and BW:PW ratio (C)**. Data is collated from previously published studies within our laboratory (Ditchfield et al., 2010; Hayward et al., 2012; Lean et al., 2014). Dotted line denotes the 10th and 90th centile of placental weight **(A)**, birthweight **(B)** and BW:PW ratio **(C)**, respectively. *P* < 0.05 indicates a significant correlation (Spearman correlation). *N* = 60, all subjects were from an appropriate for gestational age (AGA) population, defined as >10th and <90th of individualized birthweight centiles.

System A activity per mg of protein showed a significant positive correlation with placental weight (*P* < 0.05, Figure 3A). However, this correlation was not significant when placentas below the 10th and above the 90th centile (but still within the normal weight range) for placental weight were excluded (*P* = 0.49). This suggests that, even in an AGA population, it is placentas at the lower and upper ends of their weight range that are resulting in the extremes of system A activity. There was no correlation between birthweight and system A activity per mg protein (Figure 3B). When system A activity per mg protein was multiplied up to account for total placental weight, this was positively correlated with birthweight (*P* < 0.01) consistent with the important role of system A in supporting fetal growth. The lack of correlation between system A activity per mg protein and birthweight supports the data of Harrington and colleagues who measured system A activity in MVM vesicle preparations (Harrington et al., 1999). However, it is at odds with the data by Godfrey and colleagues who reported an inverse relationship between birthweight and system A activity measured in MVM vesicles, and of Jansson who reported a positive relationship. The reasons for these disparities are currently unclear but may reflect, in part, the differing techniques used to measure system A activity. This is particularly important when considering that villous fragments retain intracellular machinery whilst MVM vesicle preparations assess uptake across a relatively pure MVM free of the complications of placental metabolism. Also, this does not explain the disparity between the Harrington and Godfrey studies (Godfrey et al., 1998; Harrington et al., 1999) which both employed MVM vesicles to measure system A activity. These studies did, however, have differences in the way that “normal” birthweight was defined; Godfrey et al. relied upon actual birthweight and organized data into weight bins for comparison, whereas Harrington et al. employed IBCs between 10th and 90th centiles and looked at a correlation across this range. The different ways of analyzing the data thus complicate direct comparisons between these studies (Harrington et al., 1999; Godfrey et al., 1998). Importantly, studies from our laboratory show that placental system A activity and BW:PW ratio were negatively related although the correlation was not statistically significant (*P* = 0.07, Figure 3C). Here again, this trend toward significance was not seen when excluding those pregnancies that had a BW:PW ratio outside the 10th and 90th centiles (a total of 8 data points). Excluding these placentas altered the *P*-value from 0.07 to 0.35, highlighting the significant effect that these sub-groups had on the rest of the overall correlation.

What is clear is that previous data represented here differs from that expected from the UK MVM vesicle studies described above (Godfrey et al., 1998; Harrington et al., 1999). This means that the actual relationship between BW:PW ratio and system A activity, particularly in non-pathological pregnancies, remains unclear.

## Discussion

Data from the multiple studies described suggest that BW:PW ratio, whilst fairly consistent across normal populations, is altered in complications of pregnancy and particularly in SGA/FGR. However, this should be tempered by the fact that these differences are most obvious when examining multiple gestational time points and that this effect lessens, but still remains in a number of studies, at term. These data also reinforce the importance of accurately measuring trimmed placental weight in studies investigating placental function.

There are data suggesting that, in a non-pathological population, placentas adapt system A transporter activity according to placental and fetal size. These data are particularly compelling in mouse models where placental nutrient transfer capacity is positively related to F:P ratio, and thus placental efficiency. Following analysis of system A data from our previous studies (Ditchfield et al., 2010; Hayward et al., 2012; Lean et al., 2014), we found little evidence of altered system A activity across the normal placental weight range in women. Additionally, the data suggesting a correlation between BW:PW ratio and system A appeared to be skewed by those pregnancies below the 10th, and above the 90th, centile within the normal range. Thus, changes in placental efficiency, as assumed by an altered BW:PW ratio, were not matched by similar alterations in system A activity as previously suggested (Harrington et al., 1999; Coan et al., 2008a). Potential reasons for the disparity between the data presented here and previous data in human (Godfrey et al., 1998; Harrington et al., 1999) have been discussed in the earlier section but may be reflective of the different techniques and the types of analyses used (correlations vs. organization of data into binned weight groups). In terms of disparities between human and mouse data, and aside from species differences in placental structure, studies in humans measure uptake of MeAIB into the syncytiotrophoblast via sodium-dependent mechanisms. The studies in mice measure unidirectional maternofetal clearance of MeAIB per gram placenta. This calculation is based upon fetal accumulation of MeAIB which must include a step whereby MeAIB is transferred, by as yet undetermined mechanisms, across the fetal facing basal plasma membrane of the trophoblast. Additionally, the mouse studies will reflect both sodium-dependent and –independent mechanisms of MeAIB transport. These differences in what is being measured should thus be considered when comparing across species.

An important point to make is that system A is only one of a large number of nutrient transporter systems within the placenta; further studies investigating other systems in detail will be necessary to gain a more complete picture regarding the presence or absence of placental adaptation, in terms of nutrient transport, in normal and pathological populations. These systems may include other amino acid transport systems shown to be dysregulated in FGR such as system L (Jansson et al., 1998)and TauT (Roos et al., 2007) but also other important nutrients such as glucose and calcium whose placental transport has yet to be extensively characterized across the normal birthweight range in human pregnancy. Additionally, nutrient sensing pathways such as the mechanistic target of rapamycin (mTOR) pathway, important in matching placental amino acid transport to maternal nutrient availability and downregulated in FGR (Roos et al., 2007, 2009a,b), are worthy of investigation with regards to placental adaptation.

With regards to the investigation of the extremes of BW:PW ratio, an obvious but important point is that BW:PW ratio alone only offers limited insight; it is crucial to determine whether it is the placenta, or fetus, or both, that lie within the extremes (<10^th^, >90^th^). This affords greater insight into the likelihood of abnormal placental function in terms of nutrient transfer.

In terms of the ability of BW:PW ratio to detect those pregnancies at risk of complications, particularly FGR, the evidence suggests that low BW:PW ratio appears more predictive of abnormal fetal growth in pre-term populations and that its accuracy as a predictor at term may be diminished. However, in order for interventions in pre-term populations, there is a necessity for more accurate assessments of, in particular, placental size *in utero* (Higgins et al., 2013).

BW:PW ratio has been mooted as a predictor of long-term health (Barker et al., 1990) and, where morphological and functional placental adaptation occurs, these adaptations will affect the composition of the developing baby (Coan et al., 2008a). It has been suggested that these adaptations *in utero*, and the interplay between placental supply of nutrients and fetal nutrient demand, will ultimately play a role in an individual's ability to deal with physiological challenges throughout their life (Sibley et al., 2010). Further studies dissecting the physiological basis for alterations in BW:PW ratio will therefore be very important in understanding the basis of *in utero* programming.

In conclusion, the concept of BW:PW ratio as a marker of placental nutrient transfer efficiency is strongly supported by studies in mice, with clear evidence of placentas adapting their nutrient transfer capacity according to their size. In humans, the data are less conclusive in an AGA population with respect to the system A transporter and further studies are required that investigate a range of nutrients and transport systems. This knowledge would aid our understanding of whether placental adaptation, in terms of nutrient transfer, occurs in humans, and whether reduced placental nutrient transfer observed in FGR/SGA is indeed as a result of failed adaptation.

## Author contributions

CH and SL, MW, SG, and MD made substantial contributions to the acquisition, analysis and interpretation of data. CS, RJ, SG, and MD made substantial contributions to the conception and design of the work. CH and MD drafted the work. SL, CS, RJ, MW, SG, and MD revised the work critically and provided important intellectual content. CH, SL, CS, RJ, MW, SG, and MD approved the final version to be published and agree to be accountable for ensuring the accuracy and integrity of any part of the work.

## Funding

This work was supported by Tommy's - The Baby charity. MD is supported by a Career Development award from the Medical Research Council (Grant number MR/K024442/1).

### Conflict of interest statement

The authors declare that the research was conducted in the absence of any commercial or financial relationships that could be construed as a potential conflict of interest.
